# Seizure due to Lamotrigine Underexposure During High‐Volume CRRT for Hyperammonaemia: A Case Report

**DOI:** 10.1155/crcc/9950547

**Published:** 2026-09-22

**Authors:** Michael C. van Herwerden, Jan H. Elderman, Laurent M. A. Favie, Margreet Wagenmakers

**Affiliations:** ^1^ Department of Intensive Care Medicine, Erasmus MC, University Medical Center, Rotterdam, the Netherlands, bvdaihoc.com.vn; ^2^ Department of Clinical Pharmacy, Erasmus MC, University Medical Center, Rotterdam, the Netherlands, bvdaihoc.com.vn; ^3^ Department of Internal Medicine, Center for Lysosomal and Metabolic Diseases, University Medical Center, Rotterdam, the Netherlands, bvdaihoc.com.vn

**Keywords:** case report, continuous renal replacement therapy (CRRT), lamotrigine, pharmacokinetics, therapeutic drug monitoring, urea cycle disorders (UCDs)

## Abstract

**Background:**

Hyperammonaemic decompensation in urea cycle disorders (UCDs) is a medical emergency that requires prompt intervention. Continuous renal replacement therapy (CRRT) is an effective strategy for acute ammonia clearance, but it may significantly alter the pharmacokinetics of administered drugs.

**Case Presentation:**

A woman in her 40s with argininosuccinate lyase (ASL) deficiency and long‐standing, well‐controlled epilepsy treated with lamotrigine presented with a hyperammonaemic crisis and was treated with high‐volume continuous venovenous haemodialysis (CVVHD). She developed a generalised tonic–clonic seizure shortly after cessation of CVVHD. Serum lamotrigine declined from 12.7 to 6.0 mg/L over approximately 12 h, corresponding to an apparent elimination half‐life of 11.5 h. Lamotrigine was measurable in the effluent (2.9 mg/L), giving a saturation coefficient of 0.48 and an extracorporeal clearance of 38.4 mL/min during the high‐volume phase. The seizure was attributed to the abrupt underexposure of lamotrigine in a previously seizure‐free patient.

**Conclusion:**

This case underscores the clinical significance of extracorporeal clearance of antiseizure medications during CRRT in patients with UCDs. Drug selection should consider both ammonia‐modifying and dialysability profiles. Therapeutic drug monitoring and pre‐emptive dose adjustment, or the addition of a second intravenously available agent, should be considered when high‐volume dialysis is initiated in a patient receiving a dialysable antiseizure medication.

Learning Points

• High‐volume CRRT is effective for rapid ammonia reduction in patients with UCDs.
• Lamotrigine is dialysable, and high‐volume dialysis can acutely reduce serum concentrations to subtherapeutic levels.
• In UCDs, valproate is contraindicated due to its potential to disrupt the urea cycle, whereas dialysable antiepileptic agents carry a risk of underexposure during dialysis.
• Drug selection in UCDs must account for both ammonia‐modulating properties and dialysability.
• If dialysable drugs are used, therapeutic drug monitoring is essential to prevent treatment failure due to subtherapeutic levels, together with a plan for parenteral cover during high‐volume dialysis.

## 1. Background

Urea cycle disorders (UCDs) are rare inherited metabolic diseases caused by mutations in the genes encoding the six hepatic enzymes required for nitrogen detoxification. Impaired conversion of neurotoxic ammonia into urea leads to life‐threatening hyperammonaemic crises, which may present at any age and are triggered by catabolic stress, protein overload or certain medications [1, 2]. Ammonia readily crosses the blood–brain barrier and is metabolised by astrocytes into glutamine, inducing osmotic shifts, astrocytic swelling and ultimately, cerebral oedema [3]. Standard management comprises intravenous glucose and lipids to suppress catabolism, protein restriction, arginine and citrulline supplementation and nitrogen scavengers; in severe cases, extracorporeal ammonia clearance with continuous renal replacement therapy (CRRT) is required [1].

Argininosuccinate lyase (ASL) deficiency is the second most common UCD [4, 5]. In addition to hyperammonaemic crises, patients with UCDs frequently exhibit intellectual and motor impairment and an increased risk of epilepsy [4]. Treating epilepsy in this population is challenging because valproate is contraindicated: It inhibits N‐acetylglutamate synthase and further compromises ureagenesis [6].

CRRT itself may compromise antiseizure medication exposure. A recent narrative review of antiseizure medication dosing in the intensive care unit (ICU) states that lamotrigine requires no dose adjustment during CRRT, while simultaneously noting that this recommendation rests on limited or absent data and expert opinion. However, effluent rates above 3 L/h may produce supraphysiological clearance of susceptible drugs [7]. We report a case in which high‐volume continuous venovenous haemodialysis (CVVHD) for hyperammonaemia precipitated a breakthrough seizure through lamotrigine underexposure, and we quantify the contribution of extracorporeal clearance.

## 2. Case Presentation

### 2.1. Symptoms

A woman in her 40s (height: 173 cm, weight: 84 kg) with a history of ASL deficiency and long‐standing neurocognitive impairment presented to the emergency department (ED) after 1 day of nausea and poor oral intake. She had one episode of vomiting, which contained undigested food from the previous evening, suggesting delayed gastric emptying. The patient resides in a supported living facility, where she adheres to a protein‐restricted diet and follows her prescribed medication regimen. According to her caregivers, she had become noticeably less verbal and appeared confused compared with her baseline functioning over the past few hours.

Her medical history included well‐controlled epilepsy, managed with lamotrigine at a dose of 225 mg twice daily. She had been treated with lamotrigine since 2014. She had been seizure‐free for several years, with therapeutic drug monitoring consistently showing lamotrigine levels between 10–14 mg/L (laboratory reference range: 2.5–15 mg/L). The last oral dose before presentation was administered at 08:00 on the day of admission. Upon examination, the patient was somnolent and responded minimally to verbal stimuli. Arterial blood gas analysis revealed mild respiratory alkalosis, and her cardiovascular parameters were within normal limits.

Initial laboratory investigations revealed a plasma ammonia level of 381 *μ*mol/L (reference < 45 *μ*mol/L). Urea was 0.8 mmol/L (2.5–7.5 mmol/L), as commonly seen in UCDs due to impaired hepatic ureagenesis. Creatinine was 34 *μ*mol/L (55–90 *μ*mol/L). Liver enzymes were mildly elevated (AST 70 U/L [< 31 U/L], ALT 111 U/L [< 34 U/L], LDH 303 U/L [< 247 U/L], GGT 39 U/L [< 38 U/L], ALP 159 U/L [39–98 U/L]), and inflammatory markers were low (C‐reactive protein 2.8 mg/L [< 10 mg/L], leukocytes 8.0 × 10^9^/L [3.5–10.0 × 10^9^/L]). A chest radiograph showed no signs of pulmonary infection.

No acute electroencephalogram (EEG) or brain magnetic resonance imaging (MRI) was performed during this admission; both had been obtained previously in routine neurological follow‐up. Brain MRI in 2021 showed no structural abnormality apart from mild punctiform white‐matter changes (Fazekas Grade 1), unchanged compared with 2020. Serial EEGs in 2020, 2022 and February 2025 consistently demonstrated interictal epileptiform abnormalities without electrographic seizures, with a marked increase in their index in the most recent recording, obtained 2 months before this admission.

### 2.2. Treatment

Treatment was immediately initiated in the ED and included the administration of intravenous glucose (10% at 2 L/day) to suppress catabolism. Within 90 min, full‐dose nitrogen scavengers, sodium benzoate and sodium phenylacetate (Ammonul, 20/20 g/day, with an initial bolus of 250 mg/kg of each), were administered to promote alternative nitrogen excretion. To enhance urea cycle function, an intravenous bolus of arginine (8 g over 90 min) was given, followed by a maintenance dose of 100 mg/kg per day. Levocarnitine (1 g three times daily) was also initiated, and Intralipid (soya‐oil, 200 mg/mL, 500 mL/day) was started to further mitigate catabolism.

Despite treatment, the ammonia level increased to 414 *μ*mol/L within 90 min. The clinical presentation was considered to be progressive hyperammonaemic encephalopathy due to metabolic decompensation in the setting of ASL deficiency, most likely triggered by a gastrointestinal infection. Given the risk of cerebral edema, the patient was admitted to the ICU for additional CRRT treatment, which was initiated approximately 90 min after admission.

CVVHD was performed using the multiFiltratePRO system, equipped with an AV1000 polysulfone filter (Fresenius Medical Care, Bad Homburg, Germany), the standard filter in our unit. High‐volume dialysis was carried out using MultiBic K4 (potassium 4 mmol/L) dialysate (Fresenius Medical Care), with a blood flow of 200 mL/min and dialysate flow of 4800 mL/h, resulting in an effluent rate of 57 mL/kg/h. Anticoagulation was achieved with intravenous epoprostenol according to local protocol.

CVVHD was preferred because ammonia is a small, unbound molecule that is cleared efficiently by diffusion, and the highest feasible blood and dialysate flows were used because its clearance is flow‐dependent. In the absence of adult‐specific guidance, the ammonia target was extrapolated from paediatric consensus recommendations: below 150 *μ*mol/L and ideally 80–100 *μ*mol/L [8].

After 6 h, due to a favourable decline in plasma ammonia levels, the intensity of CVVHD was reduced to a standard protocol: blood flow of 100 mL/min, dialysate flow of 2000 mL/h, corresponding to an effluent rate of 24 mL/kg/h. Ammonia levels continued to decline, and CRRT was discontinued 8 h and 20 min after initiation.

Shortly after cessation of CRRT, the patient experienced a generalised tonic–clonic seizure. She was treated with intravenous midazolam (10 mg) and a total of 150 mg of propofol (administered in three divided doses of 50 mg) due to ongoing seizure activity. Apart from mild hypophosphatemia (0.71 mmol/L [0.84–1.50 mmol/L]), no electrolyte disturbances were noted (potassium 3.7 mmol/L [3.5–5.1 mmol/L], magnesium 0.80 mmol/L [0.70–1.05 mmol/L], sodium 138 mmol/L [136–145 mmol/L]). Neurological evaluation revealed no focal deficits, and recovery from the postictal phase was normal. Given the absence of focal neurological signs, the full recovery and the availability of recent structural and EEG characterisation, a CT scan of the brain was deemed unnecessary. Continuation of her existing AED with lamotrigine was advised by the neurology consultant.

**Figure 1:**
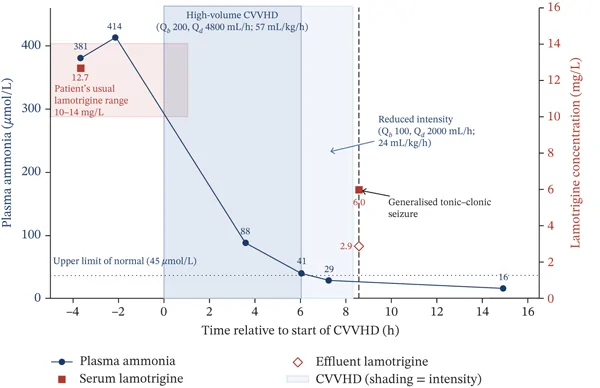
Time course of plasma ammonia and lamotrigine relative to the start of continuous venovenous haemodialysis (CVVHD; T = 0). Dark shading denotes the high‐volume phase (blood flow 200 mL/min, dialysate flow 4800 mL/h; 57 mL/kg/h), and light shading denotes the reduced‐intensity phase (100 mL/min, 2000 mL/h; 24 mL/kg/h). The red band is the patient′s usual lamotrigine range (10–14 mg/L), and the dotted line is the upper limit of normal for ammonia (45 *μ*mol/L). The open diamond is the effluent lamotrigine concentration.

Given the timing of the seizure, subtherapeutic lamotrigine exposure was suspected. Retrospective analysis of previously collected blood samples revealed a decline in serum lamotrigine concentration from 12.7 mg/L at presentation (just prior to CRRT initiation) to 6.0 mg/L at the time of the seizure. The final effluent sample contained a lamotrigine concentration of 2.9 mg/L, corresponding to a saturation coefficient of 0.48. The evening dose of lamotrigine on the day of admission was withheld because of the depressed level of consciousness and delayed gastric emptying; lamotrigine is available only as an oral formulation. The morning dose on the following day was administered. Table 1 and Figure 1 summarise the time course of plasma ammonia and lamotrigine levels alongside key therapeutic interventions and clinical events.

**Table 1:** Time course of ammonia and lamotrigine levels with associated interventions and clinical events.

Time (relative to CVVHD start)	Day	Ammonia (*μ*mol/L)	Lamotrigine serum (mg/L)	Lamotrigine effluent (mg/L)	Therapy administered	Event
T = –3:40	0	381	12.7	—	Start IV glucose 10%	—
T = –2:09	0	414	—	—	Start Ammonul 40 g/day	—
T = 0:00	0	—	—	—	—	Start CVVHD (Qb 200, Qd 4800)
T = +3:35	1	88	—	—	—	CVVHD and metabolic therapy ongoing
T = +6:03	1	41	—	—	—	CVVHD settings reduced (Qb 100, Qd 2000)
T = +7:15	1	29	—	—	—	—
T = +8:20	1	—	—	—	—	CVVHD stopped upon receiving ammonia result from T = +7:15
T = +8:35	1	—	6.0	2.9	IV midazolam (10 mg) and propofol (150 mg) given	Seizure
T = +14:54	1	16	—	—	Lamotrigine continued	

*Note:* T = 0 corresponds to the start of CVVHD.

Abbreviations: CVVHD, continuous venovenous haemodialysis; Qb, blood flow rate; Qd, dialysate flow rate.

### 2.3. Outcome

The generalised tonic–clonic seizure was attributed to subtherapeutic lamotrigine levels following significant extracorporeal clearance during high‐volume CRRT. Given the patient′s underlying epilepsy and recent hyperammonemic crisis, the abrupt decline in drug levels likely precipitated seizure activity in a brain with a lowered seizure threshold. No other cause of the seizure was identified upon further investigation.

The patient made a full neurological recovery over the following days. Protein intake was gradually reintroduced, and the acute metabolic crisis protocol was successfully tapered. To prevent recurrence during future CRRT sessions, lamotrigine was continued, and an emergency plan was established to administer intravenous levetiracetam loading during future high‐volume CRRT sessions. No further episode requiring CRRT has occurred since, so this plan has not yet been executed. No follow‐up lamotrigine concentration was obtained after the morning dose on the day following the seizure. The patient was discharged in good clinical condition, with follow‐up coordinated through her metabolic care team.

## 3. Discussion

Hyperammonaemia in UCDs is a medical emergency that requires prompt intervention to prevent irreversible neurological injury. CRRT, in conjunction with standard treatments such as high‐dose nitrogen scavengers, is a well‐established and effective approach for rapid ammonia reduction in acute decompensations [1, 8]. In this case, high‐volume CRRT resulted in an over 85% reduction in plasma ammonia levels within 6 h, which is consistent with previously reported outcomes [9].

Although CRRT is lifesaving, it can significantly alter the pharmacokinetics of drugs with low to moderate protein binding and a limited volume of distribution, such as antiseizure medications [10, 11]. The magnitude of removal during CVVHD can be estimated as the product of the delivered effluent rate and the saturation coefficient, which is the ratio of the drug concentration in the dialysate to that in plasma [10]. Table 2 summarises the dialysability of various AEDs during a 4‐h session of intermittent haemodialysis and whether they are contraindicated in UCDs [7, 10, 12–14].

**Table 2:** Dialysability and allowed use of antiepileptic drugs in UCDs. Dialysable fractions relate to a 4‐h session of intermittent haemodialysis and are indicative rather than directly transferable to continuous modalities.

Antiepileptic drug	Dialysability	Dialysable fraction (%) during 4 h of iHD	Allowed in UCDs	Reference
Levetiracetam	Good	60%–70%	Yes	[7, 12, 13]
Gabapentin	Good	35%–65%	Yes	[12, 13]
Pregabalin	Good	50%–70%	Yes	[12, 13]
Zonisamide	Good	~50%	Yes	[12, 14]
Lamotrigine	Moderate	20%–30%	Yes	[12, 13, 15]
Topiramate	Moderate	20%–30%	Yes	[7, 12]
Lacosamide	Moderate	20%–40%	Yes	[7, 12]
Carbamazepine	Poor	< 5%	Yes	[10, 12]
Valproate	Poor	< 10%	No (contraindicated)	[6, 10, 12]
Phenytoin	Poor	< 5%	Yes	[11, 12]
Clobazam	Poor	< 5%	Yes	[7, 12]

Lamotrigine is primarily metabolised by hepatic glucuronidation, with only 2%–10% excreted unchanged in urine [16]. However, due to its physicochemical properties, approximately 55% protein binding and a volume of distribution around 1.1 L/kg, it is susceptible to extracorporeal removal during dialysis [7, 12].

Serum lamotrigine fell from 12.7 to 6.0 mg/L over 12 h and 26 min, a decline of 53% corresponding to an apparent elimination half‐life of 11.5 h, against a reported half‐life of approximately 30 h during monotherapy [7, 17, 18]. Assuming intrinsic clearance alone, with the evening dose withheld but no extracorporeal removal, the predicted concentration at the time of the seizure would have been approximately 9.5 mg/L. The measured saturation coefficient of 0.48 is closely concordant with the value of 0.45 predicted from a protein‐bound fraction of 55% [10], supporting the validity of the measurement. Applying this coefficient to the prescribed dialysate flow yields an extracorporeal clearance of 38.4 mL/min (27.4 mL/kg/h) during the high‐volume phase and 16.0 mL/min (11.4 mL/kg/h) after intensity reduction, against an intrinsic clearance estimated at approximately 30 mL/min (26–36 mL/min) [17, 18]. Extracorporeal removal therefore more than doubled total lamotrigine clearance.

It should be acknowledged that a concentration of 6.0 mg/L lies within the population reference range for lamotrigine of 2.5–15 mg/L [7, 14]. However, this patient had been maintained at 10–14 mg/L for years and had been seizure‐free on that exposure. Exposure–response relationships for lamotrigine are poorly defined, and individual patients differ considerably in the concentration required for seizure control [7, 14].

Most published data on lamotrigine dialysability derive from overdose scenarios or from intermittent haemodialysis in patients with chronic kidney disease, in which approximately 17%–30% of drug is removed per session and the half‐life is shortened by roughly 75% during dialysis [15, 19, 20]. Data on antiseizure medication clearance during continuous modalities are sparse and consist largely of case reports [12, 13]. The most recent intensive care guidance recommends no dose adjustment for lamotrigine during renal replacement therapy of any intensity, while explicitly flagging that this recommendation is based on limited to absent data and expert opinion, and that effluent rates above 3 L/h may result in supraphysiological clearance of susceptible drugs [7].

In patients with UCDs, epilepsy is frequently present, and the selection of AEDs must consider both their potential to elevate ammonia levels and their pharmacokinetic profiles. Sodium valproate is contraindicated because it can induce hyperammonaemia by inhibiting NAGS, further impairing ureagenesis [6]. Although lamotrigine does not elevate ammonia, its dialysability presents challenges during CRRT. Additionally, lamotrigine is available only in oral form, making administration difficult in patients with altered consciousness. The need for an additional dose to anticipate increased drug clearance adds complexity, especially when gastroparesis during a crisis further complicates drug absorption, as illustrated by the withheld evening dose in our patient.

Levetiracetam, despite also being dialysable due to its low protein binding and small volume of distribution, offers a broader therapeutic window and allows for rapid intravenous titration [7, 21]. This makes it a more practical alternative, either as an add‐on or as a primary AED, in the context of patients with UCDs.

This report has limitations. Only two serum lamotrigine concentrations were available; therefore, the intervening course could not be characterised and the apparent half‐life is derived from two points. The saturation coefficient rests on a single effluent sample, and no follow‐up concentration was obtained after CRRT, so the expected recovery towards baseline could not be documented. Finally, the withheld evening dose is a codeterminant of the observed decline.

This case underscores the importance of therapeutic drug monitoring during CRRT, particularly for agents with narrow therapeutic indices and significant extracorporeal clearance. Preemptive dose adjustment or the addition of a second intravenous AED should be considered when initiating high‐efficiency dialysis to prevent subtherapeutic exposure, and a plan should be in place for oral medication that cannot be administered during a crisis. Effective multidisciplinary coordination among metabolic specialists, intensivists, neurologists and pharmacists is essential to ensure safe and effective treatment for this vulnerable patient population.

## Author Contributions

All authors contributed to the conception and design of the report, the interpretation of the clinical data and the drafting and critical revision of the manuscript.

## Funding

No funding was received for this manuscript.

## Disclosure

All authors have read and approved the final version of the manuscript. Michael C. van Herwerden, as corresponding author and manuscript guarantor, had full access to all of the data in this study and takes complete responsibility for the integrity of the data and the accuracy of the data analysis. The manuscript guarantor affirms that this manuscript is an honest, accurate and transparent account of the case being reported; that no important aspects have been omitted; and that any discrepancies from the case as originally documented have been explained.

## Consent

Written informed consent for publication of this case and any accompanying clinical information was obtained from the patient and cosigned by her parent as legal representative.

## Conflicts of Interest

The authors declare no conflicts of interest.

## Data Availability

The data that support the findings of this study are available from the corresponding author upon reasonable request, subject to the constraints of patient confidentiality.
